# Functional, thermodynamics, structural and biological studies of *in silico*-identified inhibitors of *Mycobacterium tuberculosis* enoyl-ACP(CoA) reductase enzyme

**DOI:** 10.1038/srep46696

**Published:** 2017-04-24

**Authors:** Leonardo K. B. Martinelli, Mariane Rotta, Anne D. Villela, Valnês S. Rodrigues-Junior, Bruno L. Abbadi, Rogério V. Trindade, Guilherme O. Petersen, Giuliano M. Danesi, Laura R. Nery, Ivani Pauli, Maria M. Campos, Carla D. Bonan, Osmar Norberto de Souza, Luiz A. Basso, Diogenes S. Santos

**Affiliations:** 1 Instituto Nacional de Ciência e Tecnologia em Tuberculose (INCT-TB), Centro de Pesquisas em Biologia Molecular e Funcional, Pontifícia Universidade Católica do Rio Grande do Sul, 90619-900, Porto Alegre, RS, Brazil; 2 Instituto de Toxicologia e Farmacologia, Pontifícia Universidade Católica do Rio Grande do Sul, 90619-900, Porto Alegre, RS, Brazil; 3 Faculdade de Medicina, Pontifícia Universidade Católica do Rio Grande do Sul, 90619-900, Porto Alegre, RS, Brazil; 4 Laboratório de Neuroquímica e Psicofarmacologia, Pontifícia Universidade Católica do Rio Grande do Sul, 90619-900, Porto Alegre, RS, Brazil; 5 Laboratório de Bioinformática, Modelagem e Simulação de Biossistemas – LABIO, Faculdade de Informática, Pontifícia Universidade Católica do Rio Grande do Sul, 90619-900, Porto Alegre, RS, Brazil; 6 Faculdade de Odontologia, Pontifícia Universidade Católica do Rio Grande do Sul, 90619-900, Porto Alegre, RS, Brazil; 7 Laboratório de FarmInformática - FarmInf, Faculdade de Farmácia, Pontifícia Universidade Católica do Rio Grande do Sul, 90619-900, Porto Alegre, RS, Brazil

## Abstract

Novel chemotherapeutics agents are needed to kill *Mycobacterium tuberculosis*, the main causative agent of tuberculosis (TB). The *M. tuberculosis* 2-*tran*s-enoyl-ACP(CoA) reductase enzyme (*Mt*InhA) is the druggable *bona fide* target of isoniazid. New chemotypes were previously identified by two *in silico* approaches as potential ligands to *Mt*InhA. The inhibition mode was determined by steady-state kinetics for seven compounds that inhibited *Mt*InhA activity. Dissociation constant values at different temperatures were determined by protein fluorescence spectroscopy. van’t Hoff analyses of ligand binding to *Mt*InhA:NADH provided the thermodynamic signatures of non-covalent interactions (Δ*H*°, Δ*S*°, Δ*G*°). Phenotypic screening showed that five compounds inhibited *in vitro* growth of *M. tuberculosis* H37Rv strain. Labio_16 and Labio_17 compounds also inhibited the *in vitro* growth of PE-003 multidrug-resistant strain. Cytotoxic effects on Hacat, Vero and RAW 264.7 cell lines were assessed for the latter two compounds. The Labio_16 was bacteriostatic and Labio_17 bactericidal in an *M. tuberculosis*-infected macrophage model. In Zebrafish model, Labio_16 showed no cardiotoxicity whereas Labio_17 showed dose-dependent cardiotoxicity. Accordingly, a model was built for the *Mt*InhA:NADH:Labio_16 ternary complex. The results show that the Labio_16 compound is a direct inhibitor of *Mt*InhA, and it may represent a hit for the development of chemotherapeutic agents to treat TB.

Tuberculosis (TB), caused mainly by *Mycobacterium tuberculosis*, still is one of the major threats in public health worldwide. In 2014, approximately 9.6 million people contracted TB, and the death toll was estimated as 1.5 million^1^. In addition, an estimated 320,000 of new cases were multidrug-resistant TB (MDR-TB) claiming the lives of 190,000 people, and 9.7% of resistant TB are extensively drug-resistant (XDR-TB)^1^. Drug resistance severely threatens TB control, by increasing the probability of a return to a time when drugs are no longer effective^2^. Co-infection of Mtb and human immunodeficiency virus (HIV) poses a major challenge since an impaired immune system potentiates TB infection, making the latter the main cause of death of HIV-infected patients^3^. Owing to the increasing number of drug-resistant strains, Mtb-HIV co-infection and the lengthy TB treatment (at least six months), new strategies are needed to combat TB. Ideally, a new anti-TB candidate should be more active than the existing drugs to reduce time of treatment, be effective against MDR-TB and XDR-TB, and be compatible with current anti-retroviral therapy^4^. In addition, it should not display any antagonism with other TB drugs to maintain a treatment with at least three active drugs, and be able to eradicate in different physiological stages, such as non-replicating and asymptomatic latent *M. tuberculosis*.

Enzymes of mycobacterial Type II dissociated fatty acid biosynthesis system (FAS-II) are attractive targets for the rational design of anti-TB agents. The FAS-II system elongates acyl fatty acid precursors yielding the long carbon chain (50–60 carbons) of the meromycolate branch of mycolic acids of mycobacteria^5^,^6^,^7^,^8^. Mycolics acids are high-molecular-weight α-alkyl, β-hydroxy fatty acids bound as esters to tetramycolypentaarabinosyl clusters in the cell wall^6^. These structures have been linked to virulence, to the ability of survival and replication of the bacillus inside macrophages, and as barrier for intracellular entry of a number of common antibiotics^7^. The FAS-II is absent in mammals suggesting that selective and low toxicity agents may be developed. The 2-*trans*-enoyl-ACP (CoA) reductase (*Mt*InhA; EC 1.3.1.9) protein, which is a member of the mycobacterial FAS-II system, catalyzes the hydride transfer from 4*S* hydrogen of NADH to carbon-3 of long chain enoyl thioester substrates to yield NAD^+^ and acyl-ACP(CoA) products^9^,^10^,^11^, has been shown to be the major target of isoniazid^9^,^10^, the most prescribed anti-TB agent.

We have previously employed two different virtual-ligand-screening approaches to identify *Mt*InhA inhibitors from a library of chemical compounds selected from the ZINC database^12^. In the first approach, a 3-D pharmacophore model of four points was built based on 36 available *Mt*InhA crystal structures and used to select molecules able to satisfy the binding features of *Mt*InhA substrate binding cavity^12^. The second approach consisted of using four well established docking programs, with different search algorithms, to compare the binding mode and score of the selected molecules from the aforementioned library^12^. Nineteen molecules from an initial data set of approximately of 1 million that could bind to *Mt*InhA:NADH binary complex were identified^12^. Preliminary results showed that six of these 19 compounds (three from each approach) showed some degree of *Mt*InhA inhibition^12^. The present work aims at evaluating 14 compounds from the original set of 19 compounds. The half-maximum inhibitory concentration (*IC*_50_) of *Mt*InhA enzyme activity and determination of minimum inhibitory concentration (MIC) to arrest *in vitro* growth of *M. tuberculosis* H37Rv (pan-sensitive) and PE-003 (multidrug-resistant) strains were carried out to select promising chemical compounds The *in vitro* mode of inhibition of *Mt*InhA activity by steady-state kinetics was determined for these selected chemical compounds. Thermodynamic analyses by fluorescence spectroscopy measuring the binding of seven compounds to *Mt*InhA:NADH binary complex were carried out. Cytotoxicity in mammalian cells (HaCat, RAW 264.7, and Vero cells) and in Zebrafish (*Danio rerio*) was evaluated for two compounds (Labio_16 and Labio_17). The intracellular activity of the latter compounds was also evaluated in macrophage (murine cell line RAW 264.7) infected with virulent *M. tuberculosis* H37Rv strain. We propose that the Labio_16 compound may be a lead compound for further efforts to develop anti-TB agents owing to its inhibitory activity of *Mt*InhA enzyme, spontaneous and favorable binding process, efficacy against H37Rv and PE-003 (a drug-resistant strain), intracellular activity in a macrophage model, and lack of detectable cytotoxic and cardiotoxic effects. Interestingly, Labio_16 is a drug candidate not a pro-drug as isoniazid needs to be activated by the mycobacterial catalase-peroxidase KatG to form an isonicotinyl-NAD adduct that inhibits *Mt*InhA enzyme activity^13^. This compound may also be a useful tool to improve our understanding of the biological role of *Mt*InhA inhibition in the absence of KatG activation. However, it should be pointed out that further efforts will have to be pursued to show whether or not *Mt*InhA is the molecular target of Labio_16 chemical compound.

## Materials and Methods

### Reagents

All chemicals were of analytical or reagent grade and were used without further purification, unless stated otherwise. NADH and Pipes were purchased from Sigma-Aldrich^®^, dimethyl sulfoxide (DMSO) was purchased from Merck^®^. Compounds Labio_1, Labio_6, Labio_9 and Labio_12 were purchased from Enamime Chemical Supplier^®^; Labio_2, Labio_3 and Labio_20 were obtained from ChemBridge^®^; Labio_7, Labio_11, Labio_16 and Labio_17 were purchased from Vitas-M Laboratory^®^; Labio_8, Labio_13 and Labio_15 were purchased from Ambinter^®^. Nucleodur C-18 column (250 mm × 4.6 mm, 5 μm) was purchased from Machery-Nagel^®^. Glacial acetic acid, acetonitrile, methanol and ammonium acetated were purchased from Merck^®^.

### *Mt*InhA expression and purification

The recombinant *Mt*InhA was expressed and purified as previously described^12^,^14^. The substrate DD-CoA was synthetized^14^ and purified^15^ from 2-*trans*-dodecenoic acid and coenzyme A via anhydride formation following acylation.

### *In vitro* inhibition studies by steady-state kinetics

In order to assess the relative potency of the compounds, inhibition studies were performed by steady-state kinetic studies using a UV-2550 UV/Visible spectrophotometer (Shimadzu^©^), monitoring the NADH oxidation at 340 nm (ε_β-NADH_ = 6.22 M^−1^ cm^−1^), in the forward direction. Experiments were performed at 25 °C, in 100 mM Pipes pH 7.0 and were started with the addition of the 2.2 μM *Mt*InhA to a total reaction volume of 500 μL, and monitoring the change in absorbance for 1 min. Before embarking on IC_50_ value measurements and determination of the mode of inhibition of chemical compounds, it is of paramount importance to show that inhibition, if any, is not time dependent. This is needed as IC_50_ and classical competitive, non-competitive and uncompetitive inhibition modes follow a rapid equilibrium process. Accordingly, *Mt*InhA (2.2 μM) was pre-incubated with inhibitor (10 μM), aliquots were taken at different times and added to the reaction mixture containing NADH (60 μM) and DD-CoA (45 μM), and initial velocity measurements were plotted as a function of time of pre-incubation^6^. Control experiments were carried out pre-incubating *Mt*InhA (2.2 μM) with DMSO (5%) in the absence of inhibitors, and initial velocity measured as a function of time of pre-incubation^6^.

The *IC*_50_ value, which defines the concentration of inhibitor required to half-saturate the enzyme population, was calculated for each compound. The maximal rate for the enzyme reaction was determined in the absence of inhibitor, in the presence of fixed non-saturating concentration of NADH (60 μM ≅ *K*_m_) and DD-CoA (45 μM ≅ *K*_m_)^10^,^11^,^14^. The reaction velocity was analyzed as the percentage of inhibition as a function of inhibitor concentration and data were fitted to Eq 1.


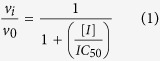


The most promising compounds were further evaluated to assess the inhibition profile and to determine the inhibition constant (*K*_i_). Initial rates were measured as a function of NADH concentration (10–160 μM) at fixed non-saturating DD-CoA concentration (45 μM) and fixed-varied inhibitor concentrations (0.5–120 μM). The *K*_i_ values towards NADH were calculated using the uncompetitive equation (Eq. 2) for compounds Labio_6 and Labio_16, in which [*I*] is the inhibitor concentration, [*S*] is the substrate concentration, *K*_m_ and *V*_max_ are, respectively, the Michaelis-Menten constant and maximum velocity, and *K*_ii_ is the overall inhibition constant for the *ESI* complex^16^. The *K*_i_ of Labio_03 was determined previously^12^.


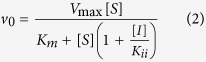


For compounds Labio_2, Labio_11, Labio_15 and Labio_17, the *K*_i_ values with respect to NADH were calculated using Eq. 3, which describes a non-competitive inhibition, where [*I*] is the inhibitor concentration, [*S*] is the substrate concentration, *K*_m_ and *V*_max_ are, respectively, the Michaelis-Menten constant and maximum velocity, *K*_ii_ is the overall inhibition constant for the *ESI* complex and *K*_is_ is the overall inhibition constant for the *EI* complex^16^.


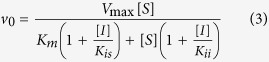


Inhibition studies were also carried out in the presence of fixed non-saturating concentration of NADH (60 μM) and fixed-varied inhibitor concentrations (0.5–120 μM), when DD-CoA was the variable substrate (15–135 μM). For compounds Labio_2, Labio_6, and Labio_16, the inhibition constants for the DD-CoA substrate were determined using Eq. 3 for the non-competitive mode of inhibition. For compounds Labio_11, Labio_15 and Labio_17, the *K*_i_ for this substrate were calculated from data fitting to the equation for competitive inhibition (Eq. 4), in which [*I*] is the inhibitor concentration, [*S*] is the substrate concentration, *K*_m_ and *V*_max_ are, respectively, the Michaelis-Menten constant and maximum velocity, and *K*_is_ is the overall inhibition constant for the *EI* complex^16^.


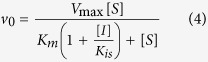


Values of the kinetic parameters and their respective errors were obtained by fitting the data to the appropriate equations by using the nonlinear regression function of SigmaPlot 9.0 (SPSS, Inc.).

### Thermodynamics of ligand binding

Binding interactions between the enzyme and ligands were evaluated by monitoring the quench in intrinsic protein fluorescence upon ligand binding using an RF-5301PC Spectrofluorophotometer (Shimadzu). The excitation wavelength was 295 nm, the emission wavelength range was 310 nm to 500 nm, excitation and emission slits were, respectively, 5 nm and 10 nm. All measurements were carried out at 15, 20, 25 and 30 °C. Fluorescence titration of pre-formed *Mt*InhA:NADH binary complex was carried out by making microliter additions of 1 mM Labio_16 (0.499–5.963 μM final concentration), 1 mM Labio_3 (0.499–8.917 μM final concentration), 1 mM Labio_6 (0.499–7.442 μM final concentration), 1 mM Labio_2 (0.499–5.963 μM final concentration), 10 mM Labio_11 (9.995–64.676 μM final concentration), 2 mM Labio_17 (0.999–17.844 μM final concentration) and 10 mM Labio_15 (4.997–89.194 μM final concentration) to 2 mL of 3 μM *Mt*InhA in the presence of 20 μM NADH, keeping the dilution to a maximum of 1%. These measurements were carried out with ligand binding to pre-formed *Mt*InhA:NADH binary complex since all compounds were shown to be able to bind to it, as borne out by their *K*_ii_ values with respect to variable concentrations of NADH in the presence of fixed-non-saturating concentration of DD-CoA (Table 1, fourth column). Even for compounds that can bind to free enzyme, the *K*_ii_ and *K*_is_ values are similar, suggesting that NADH bound to *Mt*InhA has no effect on their inhibition constants. Incidentally, the docking simulations that led to the identification of the chemical compounds here tested were carried out with NADH bound to *Mt*InhA^12^. Control experiments were employed to both determine the maximum ligand concentrations to be used with no inner filter effect and dilution effect on protein fluorescence. Data from equilibrium fluorescence spectroscopy were fitted to Eq. 5 for hyperbolic binding isotherms, in which *F*_0_ is the observed fluorescence signal, *F*_*max*_ is the maximal fluorescence intensity, *F*_∞_ is the maximum change in fluorescence at saturating ligand (L) concentration, and *K*_D_ represents the dissociation constant for binding of chemical compounds to *Mt*InhA:NADH binary complex.


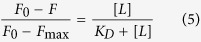


The thermodynamics binding parameters were assessed by the relationship between the equilibrium dissociation constant (*K*_D_), determined by spectrofluorimetry, and temperature. This relationship states that a change in the *K*_D_ at different temperatures yields values for changes in the enthalpy (ΔH°), in entropy (ΔS°) and in the Gibbs Free energy (ΔG°). Accordingly, *K*_D_ values were determined by fluorescence titration at 15 °C, 20 °C, 25 °C, and 30 °C. Data were fitted to the van’t Hoff Equation (Eq. 6)^17^, in which the *K*_D_ is the dissociation constant, *R* is the ideal gas constant 1.987 cal mol^−1^ K^−1^, and *T* is temperature in Kelvin, yielding ΔH° and ΔS°. An estimate for ΔG° can thus be obtained from Eq. 7.


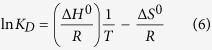



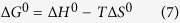


### Molecular docking protocol for building *Mt*InhA:NADH:Labio_16 ternary complex

The receptor and ligand structures were prepared using AutoDockTools^18^, while docking simulations were performed with AutoDock4.2^18^, allowing flexibility to the ligand. The docking experiment was carried out using the crystallographic structure of *Mt*InhA associated with an inhibitor (PDB ID 1P44)^19^. The 3D-grid with dimensions 90 × 60 × 60 with spacing 0.375 Å was used to limit the active site region as a search space. The Lamarckian Genetic Algorithm (LGA) was employed with 30 runs and the remaining parameters were set to their default values, except for number of evaluations, which was set to 2,500,000.

### *In vitro* Mycobacterium tuberculosis growth inhibition assay

The measurement of MIC values for each tested compound was performed in 96-well U-bottom polystyrene microplates. Isoniazid (INH, control drug) and compounds solutions were prepared at concentrations of 1 mg mL^−1^ and 4 mg mL^−1^ in neat DMSO, except Labio_3 and Labio_20 that were prepared at concentrations of, respectively, 1.8 mg mL^−1^ and 3.8 mg mL^−1^ in neat DMSO. They were diluted in Middlebrook 7H9 medium containing 10% ADC (albumin, dextrose, and catalase) to concentrations of 200 μg mL^−1^ (Labio_2, Labio_11, Labio_12, and Labio_15), 190 μg mL^−1^ (Labio_20), 90 μg mL^−1^ (Labio_3), 20 μg mL^−1^ (INH, Labio_1, Labio_6, Labio_7, Labio_8, Labio_9, Labio_13, and Labio_17), and 10 μg mL^−1^ (Labio_16) containing 5% DMSO. Serial two-fold dilutions of each drug in 100 μL of Middlebrook 7H9 medium containing 10% ADC were prepared directly in 96-well plates. Growth controls containing no antibiotic and sterility controls without inoculation were included. MIC was determined for *M. tuberculosis* H37Rv and for a clinical isolate PE-003 strains. The PE-003 strain is a multidrug-resistant clinical isolate, resistant to isoniazid, rifampicin, ethambutol, and streptomycin which contains a mutation in *inhA* regulatory region C(-15)T^20^. Mycobacterial strains were grown in Middlebrook 7H9 containing 10% OADC (oleic acid, albumin, dextrose, and catalase) and 0.05% tween 80. Cells were vortexed with sterile glass beads (4 mm) for 5 min to disrupt clumps and allowed to settle for 20 min. The absorbance of supernatant was measured at 600 nm. The *M. tuberculosis* suspensions were aliquoted and stored at −20 °C. Each suspension was appropriately diluted in Middlebrook 7H9 broth containing 10% ADC to achieve an optical density at 600 nm of 0.006 and 100 μL was added to each well of the plate except to sterility controls. The final concentration of 2.5% DMSO was maintained in each well. The plates were covered, sealed with parafilm, and incubated at 37 °C. After 7 days of incubation, 60 μL of 0.01% resazurin solution was added to each well, and incubated for additional 48 hours at 37° 21. A change in color from blue to pink indicated the growth of bacteria, and the MIC was defined as the lowest drug concentration that prevented the color change. Three tests were carried out independently, and MIC values reported here were observed in at least two experiments or were the highest value observed among the three assays.

### Cytotoxicity investigation

Cellular viability determination after incubation with the test compounds (Labio_16 and Labio_17) was performed essentially as described elsewhere^22^. Briefly, Vero (and Hacat/RAW) cells were grown in DMEM media supplemented with 10% inactivated fetal bovine serum and 1% penicillin-streptomycin. The cells were maintained in culture bottles at 37 °C in humidified atmosphere with 5% CO_2_. Cells were seeded at 3 × 10^3^ cells per well (for Hacat/RAW or Vero) in a 96-well microtiter plate and incubated for 24 hours to adhere. Medium was carefully aspirated and replaced with 90 μL DMEM, and 10 μL of stocks of the chemical compounds were added to a final concentration of 20 μM (DMSO 2.0%, v/v). After 72 h at 37 °C under 5% of CO_2_, the cultures were incubated with 3-(4,5-dimethylthiazol-2-yl)-2,5-diphenyltetrazolium bromide (MTT) (1 mg mL^−1^) for 3 h. The formazan crystals were dried at room temperature for at least 24 h and dissolved in DMSO. The absorbance was measured at 595 nm (Spectra Max M2e, Molecular Devices, USA). The percentage of cell viability for treated groups was reported considering the control wells (DMSO 0.5%-treated) as 100% of cell viability: cell viability (%) = (absorbance of treated wells/absorbance of control wells) x 100. Data are expressed as mean of cell viability ± standard error of mean of three independent experiments performed in triplicate.

### Intracellular activity investigation

To address the issue of compound activity on intracellular mycobacteria, *M. tuberculosis*-infected macrophages were used as a functional model. Virulent *M. tuberculosis* H37Rv reference strain (ATCC 27294) was cultivated as previously described^23^. The macrophage murine cell line RAW 264.7 was cultured in DMEM (Gibco) supplemented with 10% heat inactivated fetal bovine serum (FBS) and 1% Penicillin-Streptomycin at 37 °C with 5% CO_2_. Macrophage infection procedures were performed essentially as described elsewhere^24^. Briefly, macrophages were seeded in 24-well culture plates at a density of 10^5^ cells per well in DMEM medium (supplemented with 10% FBS) and incubated for 24 h at 37 °C with 5% CO_2_. The cells were then washed twice with sterile 0.9% saline solution to remove non-adherent cells. Infection of RAW 264.7 cells with *M. tuberculosis* H37Rv was performed at a multiplicity of infection of 1:1 (bacteria per macrophage) for 3 h at 37 °C with 5% CO_2_. Infected RAW 264.7 cells were washed three times with sterile 0.9% saline solution to remove extracellular bacteria and replaced with 1 mL fresh DMEM (supplemented with 10% FBS)^24^. A control group of infected macrophages without any previous treatment was lysed with 0.025% sodium dodecyl sulfate (SDS) dissolved in sterile 0.9% saline solution in the day of treatment onset; this group was named “early control”. Infected cells were then treated with the test compounds (at 5 μM) or with the positive control drugs rifampin or isoniazid (both at 5 μM) in DMEM medium. Drug solutions were prepared in DMSO, and the final concentration of DMSO in each well was 1.0%, including in the untreated control wells (named “late control”). After 5 days of incubation, each well was gently washed and the infected macrophages were then lysed with 0.025% SDS dissolved in sterile 0.9% saline solution. Lysates were serially diluted in sterile saline and plated on Middlebrook 7H10 Agar (Difco) supplemented with 10% OADC. Bacterial colony formation was registered after incubation of plates for 20 days at 37 °C. To compare cell counts, the numbers were first converted into logarithms of CFU (log_10_ CFU). Data were evaluated by one-way analysis of variance (ANOVA), followed by Bonferroni’s post-test, using GraphPad Prism 5.0 (GraphPad, San Diego, CA, USA). Differences were considered significant at the 95% level of confidence. Each concentration of drugs was tested in triplicate, and the results were expressed as the log mean numbers of bacteria per well.

### Treatment and embryo maintenance

Wild-type Zebrafish embryos were obtained from natural mating of adult *Danio rerio* bred^25^ and maintained in an automated re-circulating tank system (Tecniplast, Italy). At 2 hpf (hours post fertilization) embryos were treated with different concentrations of compounds Labio_16 and Labio_17. Both compounds were diluted in DMSO for stock solutions of 10 μM and diluted in fish water (Reverse Osmosis equilibrated with Instant Ocean Salt) to final concentrations of 1.0 μM, 3.5 μM and 7.0 μM (compound Labio_16) and 0.1 μM, 1.0 μM, 7.5 μM and 13.0 μM (compound Labio_17). Since both compounds were diluted first in DMSO, there were two control groups for each treatment, one only with fish water and the other one with the highest concentration of DMSO used in the treatments (0.035% for Labio_16 treatment and 0.065% for Labio_17 treatment).

Embryos were maintained in a 96 well plate during the 5 days of treatment inside an incubator with light-dark cycle of 14–10 hours and controlled temperature (28 °C)^25^. The solution pH and conductivity were monitored^25^. Survival and hatching efficiency were accompanied under a stereomicroscope (OlympusSZ4045) every day, as well as developmental toxicity^26^. Data for survival evaluation were analysed by Kaplan Meier survival test.

All the experiments were conducted according to the Canadian Council on Animal Care Guidelines on care and use of fish in research, teaching and testing^27^, following the Brazilian legislation (no. 11.794/08) and the Brazilian College of Animal Experimentation (COBEA)^28^. Protocols were previously evaluated and approved by the Institutional Animal Care Committee (CEUA PUCRS).

### Cardiotoxicity and cardiac evaluation

At 2 dpf (days post fertilization) and 5 dpf embryos heart frequency were quantified. For this, a single embryo per larvae was placed under a stereomicroscope (OlympusSZ4045) in petri-dishes with system water and their heart rate was monitored for 60 seconds by a blind experimenter (N = 10 in triplicates)^25^,^26^. For all procedures, temperature was kept constant at 28 °C. Data were analysed using One-way ANOVA, followed by Tukey multiple comparisons test.

### Method development for quantification of Labio_16 and Labio_17 compounds

An HPLC equipped with a quaternary pump, DAD detector, degasser, column oven and an automatic injection system was used in this set of experiments (Thermo^®^ Scientific, Sunnyvale, USA). Stock standard solutions (1 M) of Labio_16 and Labio_17 were prepared by diluting each standard in DMSO. Standard solutions were prepared by diluting the stock solution in fish water, yielding final concentrations of 0.625, 1.25, 2.50, 5.0 and 10.0 μM for Labio_16; and 0.625, 1.25, 2.50, 5.0, 10.0 and 20 μM for Labio_17 in a final volume of 0.5 mL.

Chromatographic separations were carried out using a Nucleodur C-18 column at 20 °C. The mobile phase was a 20:80% mixture of 0.1% glacial acetic acid and acetonitrile:methanol 1:1 (v/v) for Labio_16, and a mixture of 20:80% of 20 mM of ammonium acetate and acetonitrile:methanol 1:1 (v/v) for Labio_17. A flow rate of 1.5 mL min^−1^ was employed in isocratic mode, with run time of 7 minutes for Labio_16 and 12 minutes for Labio_17. The DAD detector was set at 300 nm and a full scan was continuously performed. The calibration curve for both analytes presented a correlation coefficient above 0.99.

## Results and Discussion

### *Mt*InhA enzyme inhibition assessed by steady-state kinetics

Of the initial set of 14 compounds, seven compounds showed *IC*_50_ values in the micromolar range (Table 1). The *IC*_50_ values varied from 13 μM to 87 μM. Despite their different chemical motifs, the compounds Labio_2, Labio_3, Labio_6, Labio_11, Labio_15, Labio_16 and Labio_17 inhibited the activity of *Mt*InhA enzyme (Table 1). These results are in agreement with *in silico* screening of compounds able to bind to *Mt*InhA^12^. However, compounds Labio_1, Labio_7, Labio_8, Labio_9, Labio_12, Labio_13 and Labio_20 showed no inhibitory activity towards *Mt*InhA. The *IC*_50_ screening was employed only as a preliminary test to identify enzyme inhibitors rather than rank and select the best compounds.

**Figure 1:**
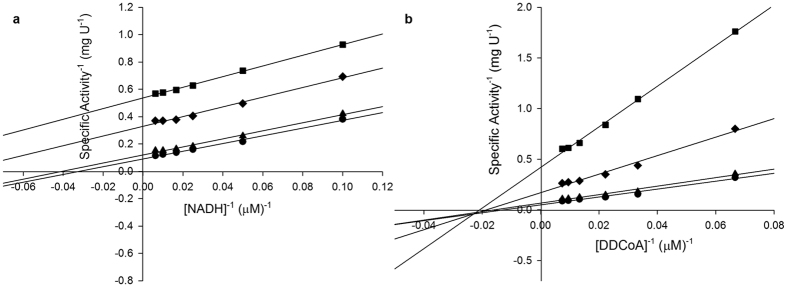
Determination of inhibition mode of Labio_16 (0–50 μM). (**a**) The Lineweaver–Burk plot displays a pattern of parallel lines, which are diagnostic of uncompetitive inhibition with respect to NADH. The data were thus fitted to Eq. 2. (**b**) The Lineweaver–Burk plot displays a pattern of lines, intersecting at the left of the *y*-axis, consistent with non-competitive inhibition mode with respect to DD-CoA. Data were fitted to Eq. 3.

Before embarking on efforts to determine the mode of inhibition of compounds selected from *IC*_50_ measurements, these compounds were evaluated to ascertain whether or not they displayed time-dependent inhibition of *Mt*InhA activity. None of them showed time-dependent enzyme inhibition (data not shown). Steady-state kinetics results showed that at varying NADH concentrations (10–160 μM) with fixed-non-saturating concentration of 2-*trans*-dodecenoyl-CoA (DD-CoA; 45 μM) the inhibition constants ranged from 1.8 to 110 μM (Table 1). For varying DD-CoA concentrations (15–135 μM) in the presence of fixed-non-saturating NADH concentration (60 μM) the inhibition constant values ranged from 0.7 to 74 μM (Table 1). For compound Labio_16, the double reciprocal plot showed a pattern of parallel lines (Fig. 1a) which is consistent with uncompetitive mode of inhibition towards NADH. Compounds Labio_3 and Labio_6 (Figure S1, Supporting Information) also displayed uncompetitive mode of inhibition with respect to NADH. This profile of inhibition indicates that the inhibitor binds exclusively to the enzyme-substrate (*ES*) complex yielding an inactive enzyme-substrate-inhibitor (*ESI*) complex^17^,^29^. Therefore, enzyme inhibition cannot be overcome by high NADH substrate concentrations. The Lineweaver-Burk plots revealed a family of lines intersecting to the left of the *y*-axis for compounds Labio_2, Labio_11, Labio_15 and Labio_17 (Figure S2, Supporting Information), indicating a non-competitive type of inhibition for these molecules with respect to NADH substrate.

The uncompetitive mode of inhibition for Labio_16, Labio_3 and Labio_6 was expected as both the pharmacophore-based approach and the virtual screening after docking simulations aimed at identifying compounds that could bind to the enoyl-thioester-substrate-binding cavity as NADH was treated as part of the protein complex (*Mt*InhA:NADH binary complex)^12^. A compound designed to bind in the substrate-binding site in the presence of NADH, acting as an uncompetitive inhibitor, would be more selective than molecules competing with the coenzyme, since many other proteins use NADH and display significant roles in different pathways. As for Labio_2, Labio_11, Labio_15 and Labio_17, the non-competitive mode of inhibition is likely due to the ability of these compounds to bind to the large enoyl thioester binding site of *Mt*InhA even in the absence of NADH. Interestingly, the binding of NADH appears to have no effect on the inhibition constant of these compounds as the *K*_ii_ and *K*_*is*_ values are fairly similar (Table 1).

When DD-CoA was the varied substrate in the presence of non-saturating concentrations of NADH, Labio_16 displayed a non-competitive type of inhibition as seen on the double-reciprocal plot (Fig. 1b). The same mode of action was found for Labio_2 and Labio_6 (Figure S3, Supporting Information) with respect to DD-CoA. Binding of DD-CoA has no effect on the inhibition constants for these compounds as *K*_ii_ and *K*_*is*_ values are similar (Table 1). Labio_11, Labio_15 and Labio_17 act as competitive inhibitors with respect to DD-CoA (Figure S4, Supporting Information) with inhibition constants in the micromolar range (Table 1). The mode of inhibition of these compounds is in agreement with *in silico* predictions^12^. On the other hand, the Labio_3 compound displayed an uncompetitive mode of inhibition with respect to DD-CoA (Table 1), which suggests that this compound binds after DD-CoA substrate has formed a complex with *Mt*InhA protein. The different modes of inhibition of the compounds here described are likely due to their chemical diversity and the large cavity of enoyl-thioester substrate binding site of *Mt*InhA^12^. Phenotypic screening was carried out for all compounds here described regardless their *IC*_50_ or *K*_i_ values. The MIC values for *M. tuberculosis* H37Rv (pan-sensitive) strain for all compounds and PE-003 (multidrug-resistant) strain for compounds Labio_16 and Labio_17 are given in Table 1.

Structure-activity relationship analysis is not warranted as the compounds here described occupy a diverse chemical space. However, some chemical features of the compounds here described are also present in other *Mt*InhA inhibitors described in the literature. Labio_2 possesses a methyl-thiazol group (Table 1) that is also present in the chemical structure of a potent *Mt*InhA inhibitor^30^. Compound 7 and analogs described elsewhere^30^, having methyl-thiazol groups, have been shown to bind to *Mt*InhA:NADH binary complex with high affinity (13.7 nM), interacting with the nicotinamide and ribose groups of NADH. This could indicate that this chemical motif is likely important to Labio_2 interaction with *Mt*InhA, and may represent a useful building block for fragment-based design of *Mt*InhA inhibitors. A thiadiazole-based compound has been shown to inhibit *Mt*InhA in the nanomolar concentration range^31^. Structural data showed that one thiadiazole ring nitrogen forms a hydrogen bond with the backbone NH of Met98 of *Mt*InhA whereas a neighbouring NH that connects two rings forms a hydrogen bond with the backbone carbonyl of Met98^31^. Interestingly, a thiadiazole ring with an adjacent nitrogen chemical motif is present in the of Labio_3 (Table 1). The Labio_3, Labio_11 and inhibitors of *Mt*InhA (Table 1) have a carbonyl-containing linker in the form of a carboxamide (Labio_2 and Labio_16), propanamide (Labio_6), urea (Labio_3) or a hydrazone-bound carbonyl (Labio_11). The importance of a carbonyl group in a series of piperazine compounds inhibitors of *Mt*InhA has been discussed^6^. Substitution of the carbonyl group for a sulfonyl moiety resulted in a 100-fold increase in the *IC*_*50*_ value, which has been proposed to be due to a reduced hydrogen-bond basicity of sulfonamide compared to amide groups^6^. However, caution should be exercised as amide groups are also present in the chemical structure of compounds Labio_1, Labio_7, Labio_9, Labio_12, and Labio_20, which failed to show any inhibitory effect on *Mt*InhA enzyme activity.

### *In vitro* Mycobacterium tuberculosis growth inhibition assays

As mentioned above, phenotypic screening was carried out for all compounds by measuring the MIC values for *in vitro* growth inhibition of *M. tuberculosis* H37Rv strain. The inhibitory effect on *in vitro* growth of PE-003 multidrug-resistant clinical isolate was determined for the compounds with the lowest MIC values. Five out of 14 compounds inhibited the growth of *M. tuberculosis* H37Rv strain, with MIC values ranging from 2.5 to 25 μg mL^−1^ (Table 1). The two lowest MIC values were for Labio_16 (2.5 μg mL^−1^) and Labio_17 (5 μg mL^−1^), which also showed inhibitory activity of *in vitro* PE-003 growth (0.6 and 2.5 μg mL^−1^, respectively). The *M. tuberculosis* PE-003 strain harbors a mutation in *inhA* regulatory region C(-15)T^20^, which appears to confer resistance to isoniazid by increasing *inhA* mRNA levels and ensuing increase in protein expression^32^. The compounds here described were *in silico* selected as ligands of *Mt*InhA protein that could be direct inhibitors of this enzyme’s activity, which would not require activation by mycobacterial KatG^12^. Further experimental data are thus needed to evaluate their activity, if any, in growth inhibition of isoniazid-resistant strains of *M. tuberculosis* harboring only *katG* structural gene mutations. At any rate, the MIC values for growth inhibition of PE-003 strain for Labio_16 (0.6 μg mL^−1^) and Labio_17 (2.5 μg mL^−1^) compounds are lower than for isoniazid (6.25 μg mL^−1^) (Table 1). Interestingly, the MIC values for *in vitro* growth inhibition of H37Rv strain for Labio_16 (2.5 μg mL^−1^ = 6.4 μM) and Labio_17 (5 μg mL^−1^ = 12.8 μM) compounds are lower than the *IC*_50_ values for Labio_16 (24 μM) and Labio_17 (20 μM) (Table 1). Similar results were obtained for the MIC values (1.5 μM for Labio_16 and 2.5 μM for Labio_17) (Table 1) for *in vitro* growth inhibition of multidrug-resistant PE-003 strain that harbors a mutation in *inhA* regulatory region C(-15)T^20^. These results might suggest that either InhA is not the target of Labio_16 and Labio_17 compounds, there may be multiple targets, or the intracellular concentration of these compounds is increased by a not yet known mechanism. Incidentally, attempts are currently underway to select for *M. tuberculosis* strains resistant to Labio_16 and Labio_17 compounds to carry out whole genome sequencing to ascertain whether or not mutations in either the regulatory region or the structural gene of *Mt*InhA occurred. A low mutation frequency would suggest multiple targets. In addition, determination of mycolic acid by thin layer chromatography shall also be carried out to provide solid evidence for InhA as the molecular target for Labio_16 and/or Labio_17 compounds.

### Thermodynamics of ligand binding

The thermodynamics parameters of binding of compounds Labio_2, Labio_3, Labio_6, Labio_11, Labio_15, Labio_16 and Labio_17 were determined by monitoring the quench in intrinsic protein fluorescence upon ligand binding at various temperatures. The van’t Hoff analysis was employed to assess the thermodynamic signatures of non-covalent interactions to each binding process. A direct analysis of ligand interactions is most appropriately carried out by isothermal titration calorimetry (ITC)^33^,^34^,^35^,^36^. However, owing to solubility issues that prevented reliable ITC data collection, fluorescence spectroscopy was employed to determine the thermodynamics of binding. The van’t Hoff analysis provides a means of determining the individual contributions of Δ*H*° and Δ*S*° to the Gibbs Free energy change (Δ*G*°) of the inhibitor binding, from the assessment of *K*_D_ as a function of temperature^34^,^36^. Table 2 gives the *K*_D_, the Δ*H*°, the Δ*S*° and the Δ*G*° parameters of ligand binding to *Mt*InhA:NADH binary complex.

**Figure 2:**
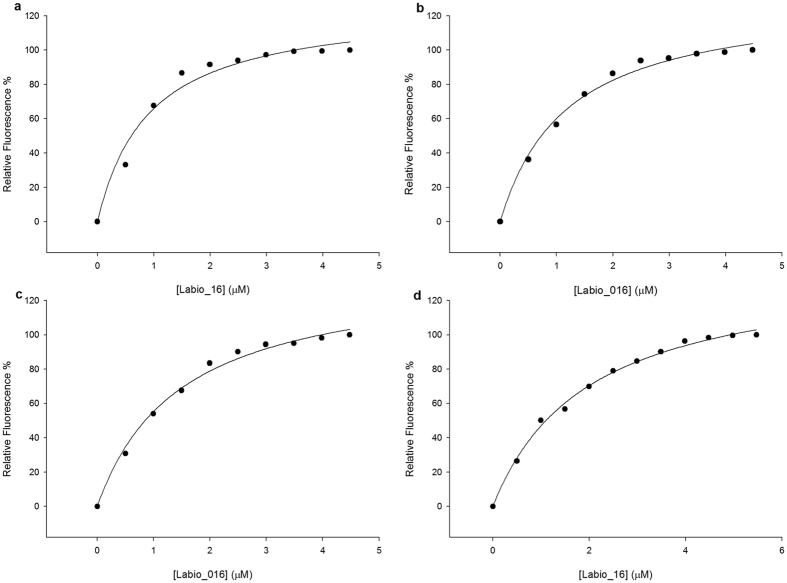
Fluorescence spectroscopy of the equilibrium binding of compound Labio_16. Changes in intrinsic protein fluorescence upon Labio_16 binding to *Mt*InhA:NADH binary complex were plotted as the relative fluorescence change as a function of increasing chemical compound concentration at 15 °C (**a**), 20 °C (**b**), 25 °C (**c**), and 30 °C (**d**).

**Figure 3:**
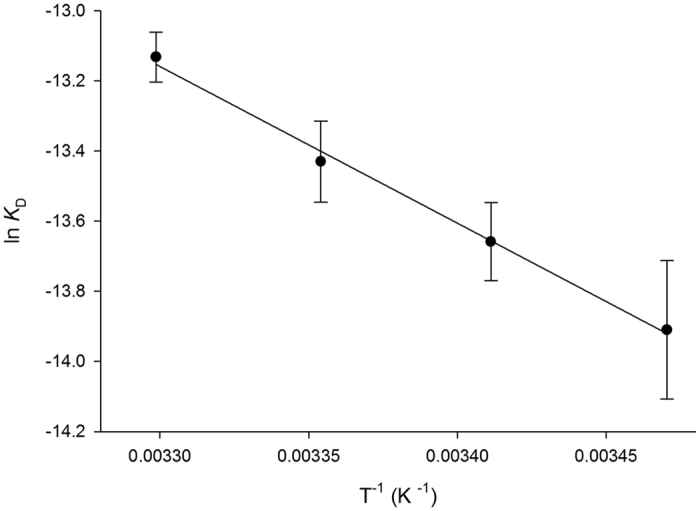
Dissociation constant as a function of temperature. The curve was fitted using the van’t Hoff equation (Eq. 6) allowing determination of ΔH° and ΔS° values. Data are expressed as the means ± SD.

Titration of *Mt*InhA:NADH complex with Labio_16 showed hyperbolic curves at all temperatures (Fig. 2), yielding *K*_D_ values of 0.9 ± 0.2 μM, 1.2 ± 0.1 μM, 1.5 ± 0.1 μM and 2.0 ± 0.1 μM at, respectively, 15 °C, 20 °C, 25 °C and 30 °C. Figure 3 shows the plot of *K*_D_ as a function of the inverse of Kelvin temperature for compound Labio_16. A linear relationship was found between the *K*_D_ and the temperature, which suggests that the Δ*H*° and Δ*S*° are independent of the temperature and isobaric heat capacity of the system (Δ*C*_p_) remained constant^33^. In addition, the hyperbolic profiles for ligand binding at all temperatures (Fig. 2) suggest that there is no cooperativity upon Labio_16 binding to *Mt*InhA:NADH binary complex. Hyperbolic profiles for ligand binding were also observed for compounds Labio_2, Labio_3, Labio_6, Labio_11, Labio_15, and Labio_17 at all temperatures, yielding their respective *K*_D_. Linear van’t Hoff plots were observed for Labio_2, Labio_3, Labio_6, Labio_11, Labio_15, and Labio_17 compounds (Figure S5). The Δ*H*° and Δ*G*° of binding for all tested compounds were negative (Table 2), indicating, respectively, exothermic reaction and spontaneous (exergonic) binding processes of inhibitors to *Mt*InhA:NADH binary complex. Compound Labio_16 binding to the latter appears to be more favorable than the other compounds tested (Δ*G*° = −8.0 kcal mol^−1^). This spontaneous process is due to a favorable enthalpic contribution (Δ*H*° = −9.4 kcal mol-1) with reduced entropic penalty (Δ*S*° = −4.8 cal mol^−1^ K^−1^). The favorable enthalpy likely originates from the changes in interatomic interactions between the *Mt*InhA:NADH binary complex and Labio_16, and the degree of the Δ*H*° depends not just on the number of interactions, but also depends on the type, length and angle of the bonds, such as hydrogen bonds or van der Waals interactions^33^,^36^. Hydrophobic interactions are related to the relative degrees of disorder in the free and bound systems and thus these interactions are reflected in the entropy change. The release of “bound” water molecules from a surface to the bulk solvent is usually a source of favourable entropy (positive Δ*S*). A reduction in conformational states in either ligand or protein upon complex formation is entropically unfavourable (negative Δ*S*)^35^. The release of water molecules from the complex to the bulk solvent would make a favorable entropic contribution, which is compensated by the unfavorable entropic contribution due to a reduction in conformational states in either the ligand or *Mt*InhA:NADH complex upon ternary complex formation. Molecular docking experiments suggested that a conformational change is needed for the inhibitor to bind to the enzyme active site in the presence of NADH^12^. The optimization of enthalpy and entropy is the clear goal of many pharmaceutical companies^36^. A straightforward strategy do improve the Gibbs energy of binding is to increase hydrophobicity of a drug candidate. However, it leads to poor solubility. Increasing the rigidity of a molecule so that upon binding no conformational restriction would ensue is also another strategy to improve entropy contribution to binding. On the other hand, improvement of binding enthalpy is more difficult to implement^36^. There exists an opportunity to improve the enthalpy of Labio_16 binding as a three-dimensional model is available which may guide the efforts of medicinal chemists. Nonetheless, crystallization trials are currently underway to obtain an experimental three-dimensional structure of *Mt*InhA:NADH:Labio_16 ternary complex to help compound optimization. Here it should be pointed out that discussion on Labio_16 compound has been favored as it has shown promising *in vitro* and *in vivo* results as shown afterwards.

The Δ*G*° of binding showed little variation for the compounds tested (Table 2: −6.2 to −8.0 kcal mol^−1^). On the other hand, larger differences were observed for Δ*H*° (−9.4 to −44.7 kcal mol^−1^) and Δ*S*° (−4.8 to −124.2 cal mol^−1^ K^−1^) (Table 2). Compounds that presented large enthalpy gains also showed large unfavorable entropic contributions, which is just another example of the enthalpy-entropy compensation phenomenon^33^,^34^,^35^,^36^. The reasoning presented above for Labio_16 compound may be extended to the other compounds given in Table 2. Since the Labio compounds share few chemical features, identification of functional groups candidates for chemical modification to optimize, preferentially, Δ*H*° of binding is not warranted.

### Computer model of *Mt*InhA:NADH:Labio_16 ternary complex

**Figure 4:**
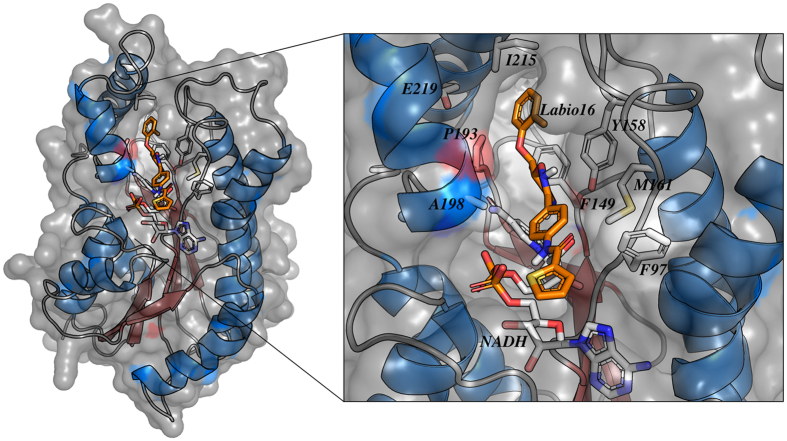
Intermolecular interactions between Labio_16 compound and *Mt*InhA:NADH binary complex. The *in silico* binding mode of Labio_16 was obtained from the docking experiment. The residues represented as sticks, including NADH, are involved in the stabilization of the compound Labio_16 in the substrate binding cavity. Image prepared with PyMOL^40^.

Docking experiment of compound Labio_16 in the active site of *Mt*InhA with NADH bound shows intermolecular interactions with the substrate binding site (Fig. 4), which is in agreement with the uncompetitive inhibition mode with respect to NADH determined experimentally (Fig. 1a). The heterocyclic 1-oxy-2,4-diazacyclopenta-2,4-diene ring of Labio_16 interacts with the side chain of Phe149 (Fig. 4). Interactions between Labio_16 and residues Phe97, Met161, Ala198, Ile215 and Glu219 can also be observed (Fig. 4). Interestingly, the furan ring of Labio_16 appears to make hydrophobic interactions with the ribose bound to the adenine of NADH (Fig. 4). It is tempting to suggest that the favourable entropic contribution from release of “bound” water molecules of hydrophobic interacting groups is counterbalanced by restrictions in conformational states of bound Labio_16 compound, resulting in a less unfavorable entropic contribution as compared to the other Labio compounds (Table 2).

### Cytotoxicity investigation

As Labio_16 and Labio_17 showed the lowest MIC values for *in vitro* growth inhibition of pan-sensitive *M. tuberculosis* H37Rv strain and the only ones active against PE-003 multidrug-resistant clinical isolate, *in vitro* cytotoxic effects of these compounds were evaluated by the MTT assay. Hacat (human keratinocytes) and Vero (African green monkey kidney) and RAW 264.7 (murine macrophages) cells were used in these experiments. Cellular viability was evaluated for these cell lineages (2000 cells/well) in the presence of Labio_16 and Labio_17 after 72 hours of incubation (Table 3). The *in vitro* incubation of these compounds, at the concentration of 20 μM, did not significantly affect cell viability of the HaCat, Vero and RAW 264.7 cell lines.

### Intracellular activity investigation

Rifampicin and isoniazid treated groups showed a decrease of 2.3 log_10_ (*P* < 0.001) and 1.7 log_10_ (*P* < 0.001) in the Colony-Forming Unit (CFU) counts compared to the untreated late control and early control groups, respectively (Table 3). Importantly, treatment with the compound Labio_17 resulted in statistically significant reductions in CFU units compared to both early and late control groups, suggesting that this compound is bactericidal (Table 3). Treatment with the compound Labio_16 resulted in statistically significant reduction in CFU counts compared to the late control. No significant difference was observed when we compared CFU loads from Labio_16-treated and early control groups, indicating that Labio_16 is bacteriostatic in this model of infection (Table 3).

### Cardiotoxicity evaluation

Efforts to try to anticipate possible side effects and/or complications due to the use of a therapeutic agent are key factors in drug discovery programs^37^. Bedaquiline, a recently TB approved drug^38^, is an example of how side effects, especially cardiac effects, can limit the use of a drug and can compromise the TB treatment. Hence, the effects of compounds Labio_16 and Labio_17 regarding cardiotoxicity, survival, hatching and heart rate were evaluated using the Zebrafish model.

**Figure 5:**
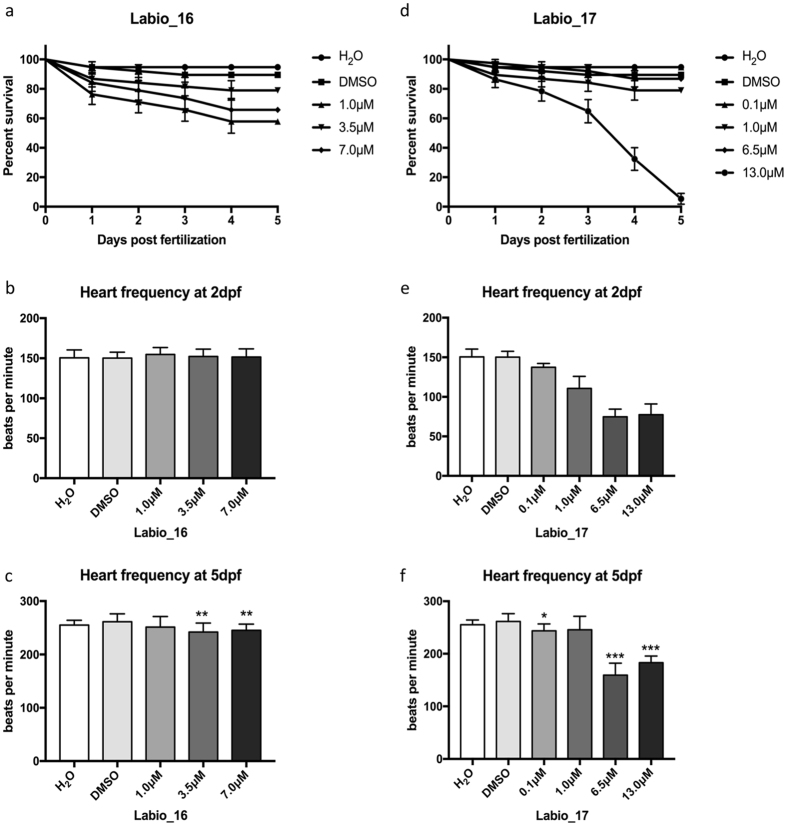
*In vivo* assay using Zebrafish embryos from 2 hpf to 5 dpf. Kaplan-Meyer survival curve was analysed during 5 days of treatment for both compounds Labio_16 (**a**) (Log-rank (Mantel-Cox) Test p = 0.0005), and Labio_17(**d**) (Log-rank (Mantel-Cox) Test p < 0.0001). Heart frequency was also evaluated at 2 dpf ((**b**), Laio_16; (**e**), Labio_17) and 5 dpf ((**c**) Labio_16; (**f**), Labio_17). Heart frequency was analysed using one-way ANOVA followed by Tukey post-hoc. (*p < 0.05 different from DMSO group; **p < 0.01 different from DMSO group; ***p < 0.001 different from DMSO and H_2_0 groups. (Graphs were plotted with means and SD).

The Labio_16 compound showed a statistical difference on survival rate (Fig. 5a) when compared to animals treated with different concentrations of Labio_16 and controls (Log-rank (Mantel-Cox) Test p = 0.0005). No difference in hatching efficiency was observed between the groups. At 3 dpf (days post fertilization) all embryos had hatched from their chorions as it was expected. Regarding the heart frequency, there was no difference between the groups at 2 dpf (Fig. 5b) using one-way ANOVA (p = 0.59; F_(4,90)_ = 0.7128). However, it was observed a statistically difference using one-way ANOVA (p < 0.001; F_(4,95)_ = 5.277) at 5 dpf (Fig. 5c). When comparing the concentrations of 3.5 μM and 7.0 μM against the DMSO control, a decrease in the heart rate (p < 0.001 and p < 0.005 respectively) was observed. The statistics for heart frequency analysis of Labio_16 using one-way ANOVA followed by Tukey post-hoc are given in Table S1.

There was a dose-dependent statistical difference on survival rate for compound Labio_17 (Fig. 5d) when compared to animals treated with different concentrations of Labio_17 and controls (Log-rank (Mantel-Cox) Test p < 0.0001). Hatching efficiency was also monitored and at the two highest concentrations (13.0 μM and 7.5 μM) a delayed hatching efficiency of most of the embryos was observed. Regarding the heart frequency, there was a significant difference between the groups at 2 dpf (Fig. 5e) using one-way ANOVA (p < 0.001; F_(5,108)_ = 202.7). In addition, when applied a Tukey post-hoc test it was observed that all groups were different when compared with the H_2_O group or the DMSO group (Table S2). At 5 dpf (Fig. 5f) it was also observed a statistically difference using one-way ANOVA (p < 0.001; F_(5,96)_ = 88.39). When comparing the concentrations of 0.1 μM and 1.0 μM against the H_2_O control, no difference was observed in the heart frequency (p = 0.35 and p = 0.58, respectively). Compound Labio_17 at 1.0 μM showed no difference when compared to the DMSO control (p = 0.08). All other groups showed a statistically difference against both controls (Table S2).

### HPLC detection of Labio_16 and Labio_17 compounds

To test whether or not the Labio_16 and Labio_17 compounds were absorbed by the embryos, liquid chromatography was employed. The developed HPLC method was also employed to both determine the amount of compounds present in the *in vivo* assay medium after an interval of 120 hours and in its stability in the same aqueous solution. A decrease of 55 ± 2% in the first 24 hours of incubation was observed for compounds Labio_16 and Labio_17 (data not shown). The stability test showed a decrease of only 21% in compound concentration (data not shown). These results show that the Labio_16 and Labio_17 compounds were indeed absorbed by the embryos.

## Conclusion

From the initial set of 14 compounds, previously identified as *Mt*InhA candidate inhibitors^12^, seven of them inhibited the *Mt*InhA enzyme activity in the *in vitro* steady-state kinetics assays. Protein fluorescence spectroscopy results showed that the binding of these seven compounds to *Mt*InhA:NADH binary complex is spontaneous (exergonic). These binding processes were dissected by van’t Hoff analysis and they showed favorable enthalpic (exothermic) and unfavorable entropic contributions. Although not all 14 compounds showed inhibition of *Mt*InhA enzyme activity, phenotypic screening was carried out for all of them by determining the MIC values for *M. tuberculosis* H37Rv strain. The compounds with the lowest MIC values for the latter strain (Labio_16 and Labio_17) also inhibited the *in vitro* growth of a multidrug-resistant clinical isolate. These two compounds showed no cytotoxic effect in Hacat, Vero and RAW 264.7 cell lines. The Labio_16 was bacteriostatic and Labio_17 bactericidal in *M. tuberculosis*-infected macrophages model. In the Zebrafish model, Labio_16 showed no cardiotoxicity whereas Labio_17 showed signs of cardiotoxicity. Accordingly, it was deemed appropriate to center the discussion of thermodynamic data and to build a model for the *Mt*InhA:NADH:Labio_16 ternary complex. The results here described suggest that the Labio_16 compound may represent a hit for further optimization phases aiming at the development of chemotherapeutic agents to treat TB. Importantly, as *Mt*InhA is a druggable *bona fide* target of isoniazid^5^,^9^ and its experimental three-dimensional structure is available^13^, a structure-based approach could also help guide optimization efforts. Notwithstanding, a number of criteria will have to be satisfied^39^ to endorse the pursuit of a hit-to-lead project. Moreover, as pointed out in the “***In vitro Mycobacterium tuberculosis*** **growth inhibition assays**” subheading, further experimental data have to be provided to ascertain whether or not *Mt*InhA is the intracellular molecular target of the chemical compounds here presented.

## Additional Information

**How to cite this article:** Martinelli, L. K. B. *et al*. Functional, thermodynamics, structural and biological studies of *in silico*-identified inhibitors of *Mycobacterium tuberculosis* enoyl-ACP(CoA) reductase enzyme. *Sci. Rep.* **7**, 46696; doi: 10.1038/srep46696 (2017).

**Publisher's note:** Springer Nature remains neutral with regard to jurisdictional claims in published maps and institutional affiliations.

## Supplementary Material

Supplementary Information

## Figures and Tables

**Table 1:** *IC*_50_ values and inhibition constants (*K*_i_) of Labio compounds on *Mt*InhA activity, and minimum inhibitory concentration (MIC) towards H37Rv and PE-003 strains of *M. tuberculosis*.

		*IC*_*50*_ (μM)	*K*_i_ (μM)				MIC (μg/ml)	
			NADH		DD-CoA		H37Rv	PE-003
			*K*_ii_^a^	*K*_is_^b^	*K*_ii_^a^	*K*_is_^b^		
INH		—	—	—	—	—	0.31	6.25
Labio_1	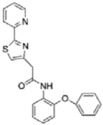	—	—	—	—	—	>10	_
Labio_2	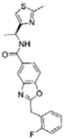	18 ± 2	2.2 ± 0.6	2.3 ± 0.8	0.7 ± 0.2	0.7 ± 0.4	>100	_
Labio_3	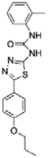	19 ± 2	24 ± 3	—	20 ± 2	—	11.25	_
Labio_6	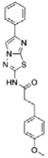	9.8 ± 0.9	1.8 ± 0.2	—	0.7 ± 0.1	0.7 ± 0.2	>10	_
Labio_7	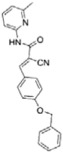	—	—	—	—	—	>10	_
Labio_8	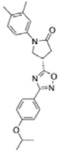	—	—	—	—	—	>10	_
Labio_9	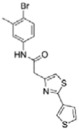	—	—	—	—	—	>10	_
Labio_11	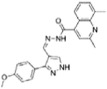	87 ± 3	41 ± 6	44 ± 14	—	27 ± 3	>100	_
Labio_12	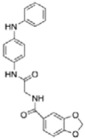	—	—	—	—	—	>100	_
Labio_13	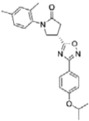	—	—	—	—	—	>10	_
Labio_15	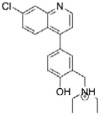	83 ± 2	110 ± 11	110 ± 26	—	74 ± 8	25	_
Labio_16	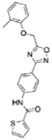	24 ± 2	7 ± 1	—	8 ± 3	13 ± 1	2.5	0.6
Labio_17	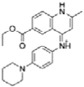	20 ± 3	8 ± 1	8 ± 3		6.3 ± 0.7	5	2.5
Labio_20	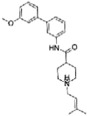	—	—	—	—	—	23.8	_

^a^*K*_ii_ is the inhibitory dissociation constant for the ESI complex.

^b^*K*_is_ is the inhibitory dissociation constant for the EI complex^23^.

**Table 2:** Dissociation constant and thermodynamics parameters for Labio chemical compounds binding to *Mt*InhA:NADH binary complex determined by fluorescence spectroscopy^a^.

	*K*_D_ (μM)	ΔH (kcal.mol^−1^)	ΔS (cal.mol^−1^.K^−1^)	ΔG (kcal.mol^−1^)
Labio_2	2.2 ± 0.2	−37.7 ± 2.7	−100.5 ± 9.2	−7.7 ± 0.7
Labio_3	4.4 ± 0.4	−27.8 ± 1.8	−68.5 ± 6.3	−7.4 ± 0.7
Labio_6	3.1 ± 0.4	−44.7 ± 3.6	−124.2 ± 12.3	−7.6 ± 0.7
Labio_11	17.9 ± 1.1	−18.7 ± 1.7	−40.9 ± 5.9	−6.5 ± 0.9
Labio_15	29.4 ± 2.1	−9.6 ± 0.8	−11.5 ± 2.8	−6.2 ± 1.5
Labio_16	1.5 ± 0.2	−9.4 ± 06	−4.8 ± 2.2	−8.0 ± 3.6
Labio_17	7.2 ± 0.5	−23.3 ± 1.9	−54.5 ± 6.5	−7.1 ± 0.8

^a^Dissociation constant determined at 298.15 K (25 °C).

**Table 3:** Data of cytotoxic effects of test compounds on HaCat, RAW 264.7 and Vero cells after 72 hours of incubation and intracellular activity against the *M. tuberculosis* H37Rv strain in infected macrophages.

Compound	% of cell viability ± SEM^§^			Mean log CFU/well ± SEM
	Vero	RAW 264.7	Hacat	
16	89 ± 12	91 ± 1	104 ± 12	3.9 ± 0.1**
17	85 ± 8	98 ± 2	84 ± 14	3.4 ± 0.1***^++^
Rifampin	—		—	2.4 ± 0.1***^+++^
Isoniazid	—		—	2.4 ± 0.1***^+++^
Early control	—		—	4.1 ± 0.1*
Late control	—		—	4.7 ± 0.1

^§^DMSO 2.0%-treated control wells were considered as 100% of cell viability. *P < 0.05 **P < 0.01 ***P < 0.001 compared to the Late control group. ^++^*P* < 0.01 ^+++^*P* < 0.001 compared to the Early control group.
