# Supplementary material for: Functional, thermodynamics, structural and biological studies of in silico-identified inhibitors of Mycobacterium tuberculosis enoyl-ACP(CoA) reductase enzyme
Source: Sci Rep. 2017 Apr 24;7:46696. doi: 10.1038/srep46696 (PMC5402281; doi:10.1038/srep46696)
Supplement: Supplementary Information [file srep46696-s1.pdf]

Supplementary Information

Functional, thermodynamics, structural and biological studies of *in silico*-identified inhibitors of *Mycobacterium tuberculosis* enoyl-ACP(CoA) reductase enzyme

Author list

Leonardo K. B. Martinelli, Mariane Rotta, Anne D. Villela, Valnês S. Rodrigues-Junior, Bruno L. Abbadi, Rogério V. Trindade, Guilherme O. Petersen, Giuliano M. Danesi, Laura R. Nery, Ivani Pauli, Maria M. Campos, Carla D. Bonan, Osmar Norberto de Souza, Luiz A. Basso and Diogenes S. Santos.

Table of contents

1. Lineweaver-Burk plots of compounds Labio_2, Labio_6, Labio_11, Labio_15 and Labio_17
2. van't Hoff plots for compounds Labio_2, Labio_3, Labio_6, Labio_11, Labio_15 and Labio_17
3. Statistical analysis of Labio_16 and Labio_17 in Zebrafish cardiotoxic model

1. Lineweaver-Burk plots of compounds Labio_2, Labio_6, Labio_11, Labio_15 and Labio_17

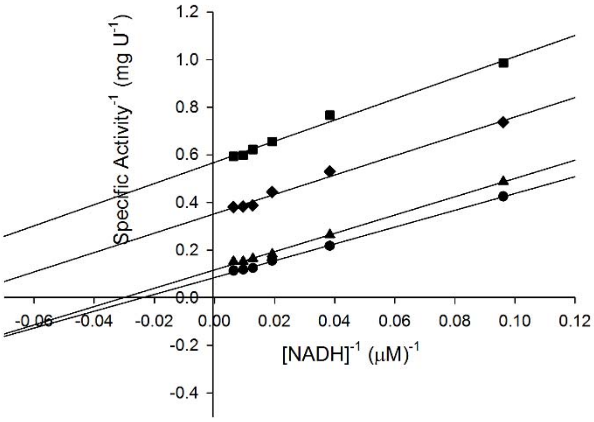

Figure S1. Double-reciprocal plot of inhibition assays for compound Labio_6 (0-20 µM). Pattern of parallel lines indicates uncompetitive inhibition towards NADH.

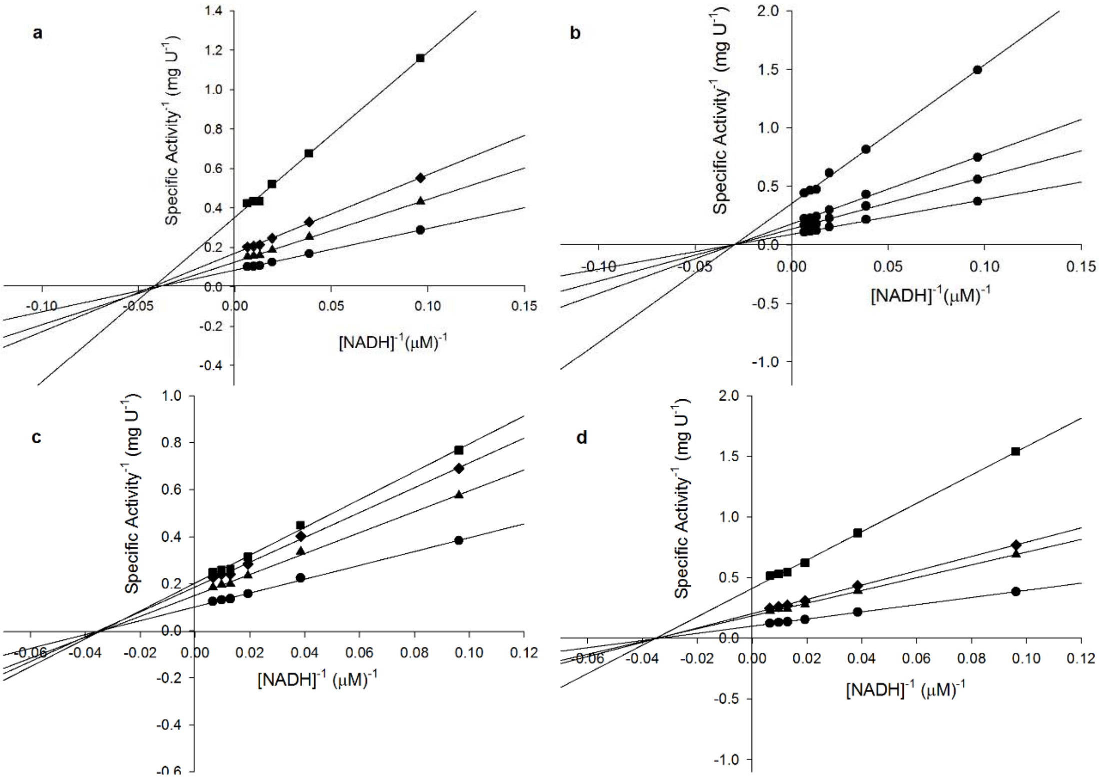

Figure S2. Double-reciprocal plots of inhibition assays: (a) compound Labio_2 (0-10 µM), (b) compound Labio_11 (0-100 µM), (c) compound Labio_15 (0-120 µM), (d) compound Labio_17 (0-20 µM). Pattern of intersecting lines at left of y-axis indicate non-competitive inhibition towards NADH.

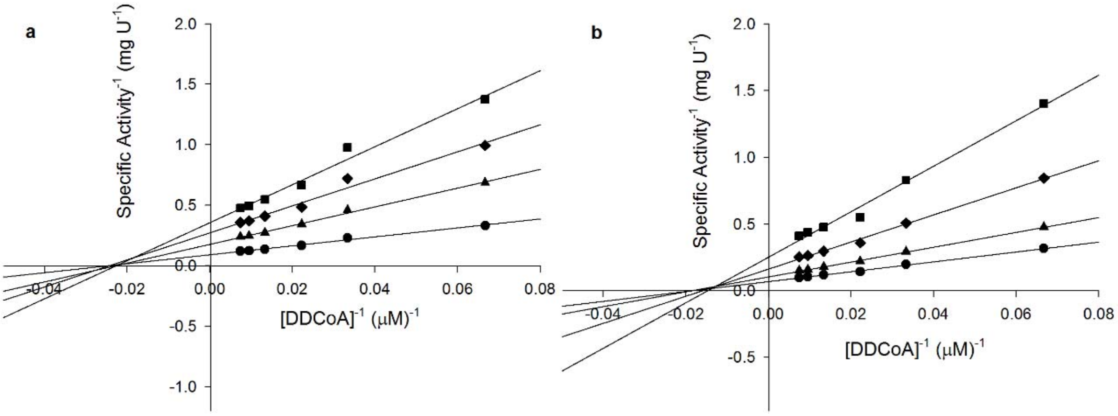

Figure S3. Double-reciprocal plots of inhibition assays: (a) compound Labio_2 (0-5 µM), (b) compound Labio_6 (0-2 µM). Pattern of intersecting lines at left of y-axis indicate non-competitive inhibition towards DD-CoA.

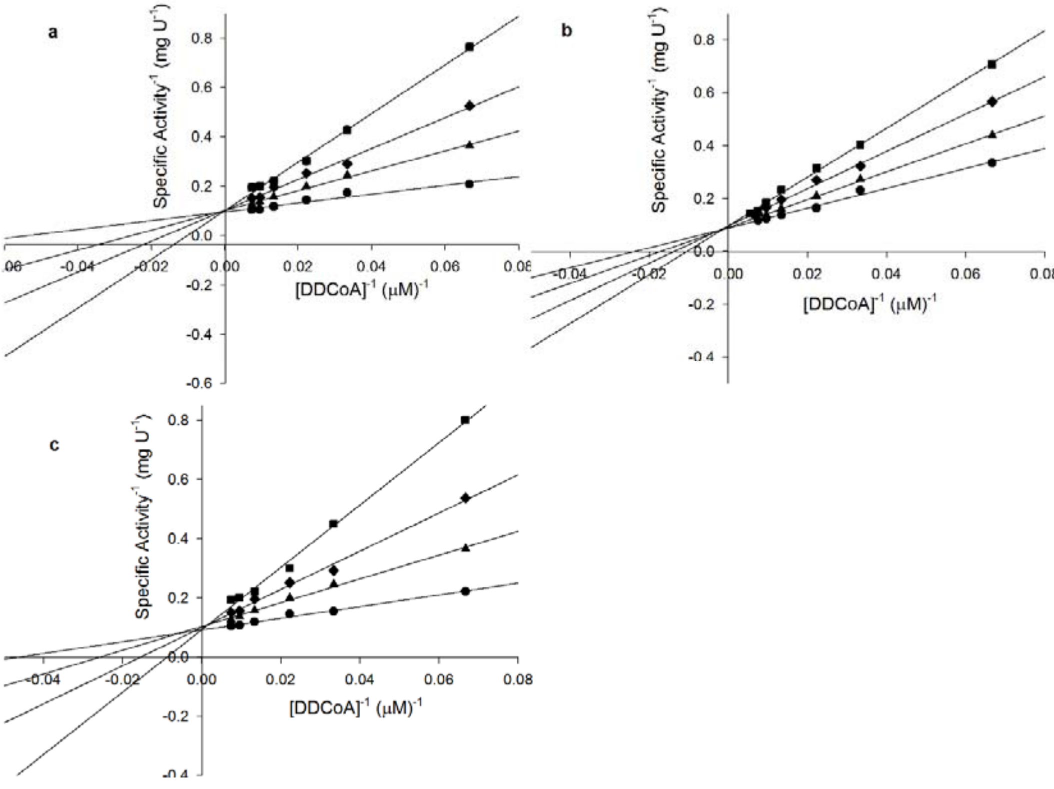

Figure S4. Double-reciprocal plots of inhibition assays: (a) compound Labio_11 (0-80 µM), (b) compound Labio_15 (0-120 µM), (c) compound Labio_17 (0-20 µM). Pattern of intersecting lines at the y-axis indicate competitive inhibition towards DD-CoA.

2. van’t Hoff plots of compounds Labio_2, Labio_3, Labio_6, Labio_11, Labio_15 and Labio_17

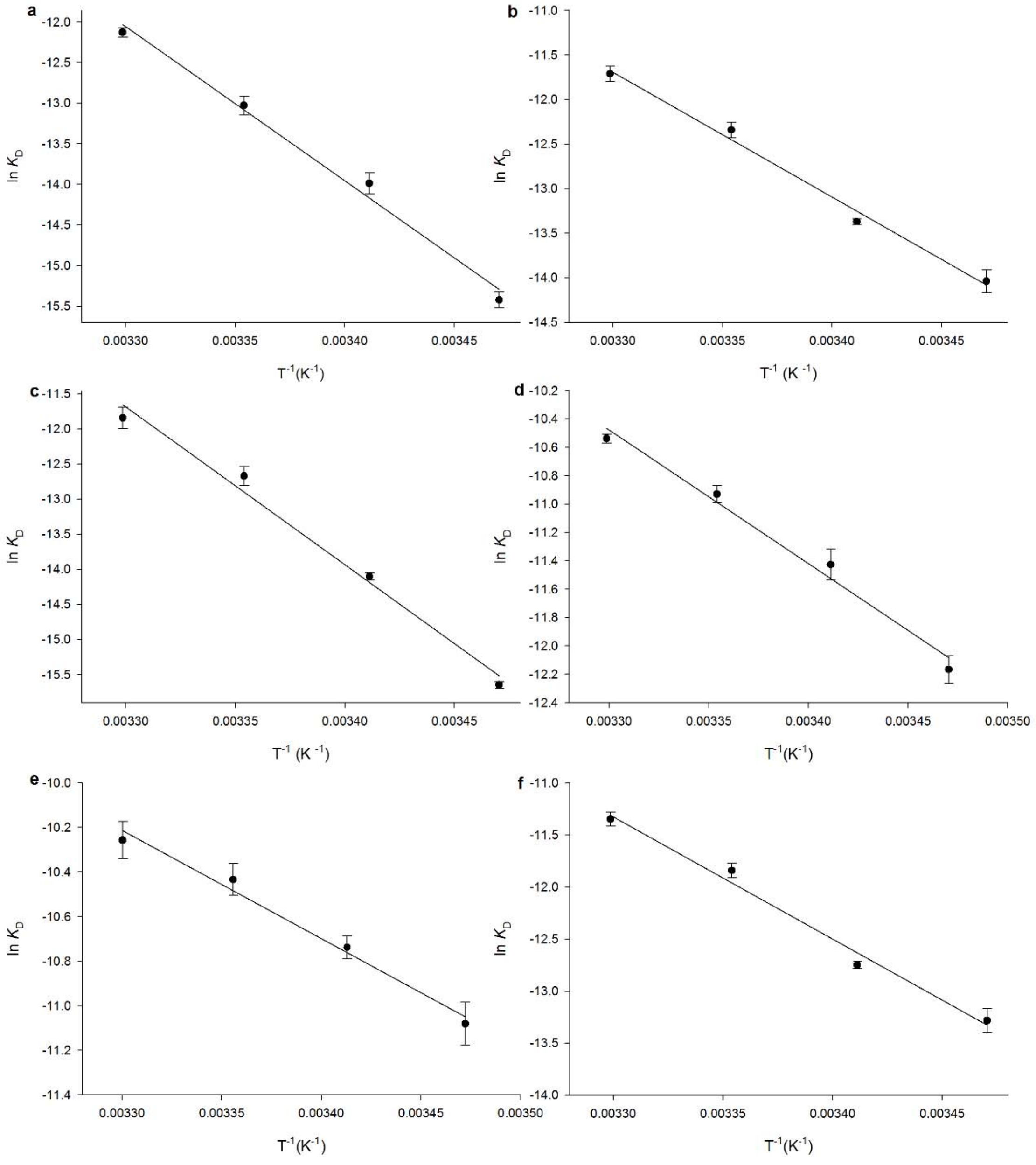

Figure S5. Dissociation constant as a function of temperature (15, 20, 25 and 30 ^{o}C) . (a) Labio_02, (b) Labio_03, (c) Labio_06, (d) Labio_11, (e) Labio_15 and (f) Labio_17. Data are expressed as the means ± SD.

3. Statistical analysis of Labio_16 and Labio_17 in Zebrafish cardiotoxic model

Table S1. Heart frequency analysis of Labio_16 using one-way ANOVA followed by Tukey post-hoc. (* $p<0.05$ different from DMSO group; ** $p<0.01$ different from DMSO group; *** $p<0.001$ different from DMSO and H_20 groups)

**Tukey's multiple comparisons test**	**justed P Value**
H_2O vs. DMSO	0.67
H_2O vs. 1.0 µM	0.94
H_2O vs. 3.5 µM	0.05
H_2O vs. 7.0 µM	0.25
DMSO vs. 1.0 µM	0.23
DMSO vs. 3.5 µM	<0.001
DMSO vs. 7.0 µM	0.009
1.0 µM vs. 3.5 µM	0.28
1.0 µM vs. 7.0 µM	0.71
3.5 µM vs. 7.0 µM	0.96

Table S2. Heart frequency analysis of Labio_17 using one-way ANOVA followed by Tukey post-hoc. (* $p<0.05$ different from DMSO group; ** $p<0.01$ different from DMSO group; *** $p<0.001$ different from DMSO and H_20 groups)

**Tukey's multiple comparisons test**	**Adjusted P Value**
H_2O vs. DMSO	0.88
H_2O vs. 0.1 µM	0.35
H_2O vs. 1.0 µM	0.58
H_2O vs. 6.5 µM	<0.001
H_2O vs. 13 µM	<0.001
DMSO vs. 0.1 µM	0.03
DMSO vs. 1.0 µM	0.08
DMSO vs. 6.5 µM	<0.001
DMSO vs. 13 µM	<0.001
0.1 µM vs. 1.0 µM	>0.99
0.1 µM vs. 6.5 µM	<0.001
0.1 µM vs. 13 µM	<0.001
1.0 µM vs. 6.5 µM	<0.001
1.0 µM vs. 13 µM	<0.001
6.5 µM vs. 13 µM	0.49
